# Concomitant Factors Leading to an Atypical Osteonecrosis of the Jaw in a Patient with Multiple Myeloma

**DOI:** 10.1155/2014/281313

**Published:** 2014-07-16

**Authors:** Jaume Miranda-Rius, Lluís Brunet-Llobet, Eduard Lahor-Soler, Josep Anton Giménez-Rubio

**Affiliations:** ^1^Departament d'Odontostomatologia, Facultat d'Odontologia, Universitat de Barcelona, L'Hospitalet de Llobregat, 08907 Barcelona, Spain; ^2^Servei d'Odontologia, Hospital Universitari Sant Joan de Déu, Universitat de Barcelona, Esplugues de Llobregat, 08950 Barcelona, Spain; ^3^Servei de Cirurgia Oral i Maxil*·*lofacial, Hospital Universitari Mútua de Terrassa, Universitat de Barcelona, Terrassa, 08221 Barcelona, Spain

## Abstract

Osteonecrosis of the jaw (ONJ) is a site specific osseous pathology, characterized by chronic exposed bone in the mouth, which needs to be reinforced periodically within the medical literature. ONJ is a clinical entity with many possible aetiologies and its pathogenesis is not well understood. The risk factors for ONJ include bisphosphonates treatments, head and neck radiotherapy, dental procedures involving bone surgery, and trauma. Management of ONJ has centred on efforts to eliminate or reduce severity of symptoms, to slow or prevent the progression of disease, and to eradicate diseased bone. This case describes a rare case of ONJ in a 64-year-old Caucasian male diagnosed with multiple myeloma stage III. The lesion was related to a traumatic injury during mastication. Eighteen months ago in the same area the molar 37 was extracted, achieving a complete satisfactory healing, when only 2 doses of zoledronic acid had been administered. *Actinomyces* bacterial aggregates were also identified in the microscopic analysis. The management of this osteonecrotic lesion included antibiotic treatment and chlorhexidine topical gel administration. The evolution was monitored every two weeks until patient's death. The authors provide a discussion of the etiology, pathogenesis, diagnosis, and management. This case report may shed light on the controversies about concomitant factors and mechanisms inducing ONJ.

## 1. Introduction

Osteonecrosis of the jaw (ONJ) is a site specific osseous pathology, characterized by exposed bone in the mouth that does not heal with 6 to 8 weeks of therapy. It is very likely that ONJ is a clinical entity with many possible aetiologies and its pathogenesis is not well understood [1]. The risk factors for ONJ include bisphosphonates treatments, head and neck radiotherapy, periodontal disease, dental procedures involving bone surgery, edentulous regions, and trauma for poorly fitting dentures [2–6].

Additional risk factors in cancer patients include the underlying malignancy, chemotherapy, corticosteroids, and systemic or regional infection [7]. ONJ has been reported in patients with a variety of tumour types, including multiple myeloma. Frequently, these patients are receiving long-term chemotherapy and many short-term intermittent corticosteroid therapies with concomitant bisphosphonate therapy for cancer and symptom management [1]. Cancer patients treated with intravenous bisphosphonates account for the majority of bisphosphonate-related ONJ cases, although individuals taking oral formulations, most often for osteoporosis, may also be affected [8].

These lesions typically become symptomatic in case of secondary infections, trauma to adjacent soft tissues, or other more rare complications such as pathologic bone fracture [1, 8].

The prevalence of ONJ is estimated to be 2–4% among cancer patients and substantially lower (0.1–0.5%) in patients taking oral bisphosphonates only [9–13]. These lesions are more frequent on the mandible than the maxilla [1].

Management of ONJ has centred on efforts to eliminate or reduce severity of symptoms, to slow or prevent the progression of disease, and to eradicate diseased bone [8].

The objective of this paper is to report an unusual clinical case of ONJ in a patient with multiple myeloma-related bone disease.

## 2. Case Report

**Figure 1:**
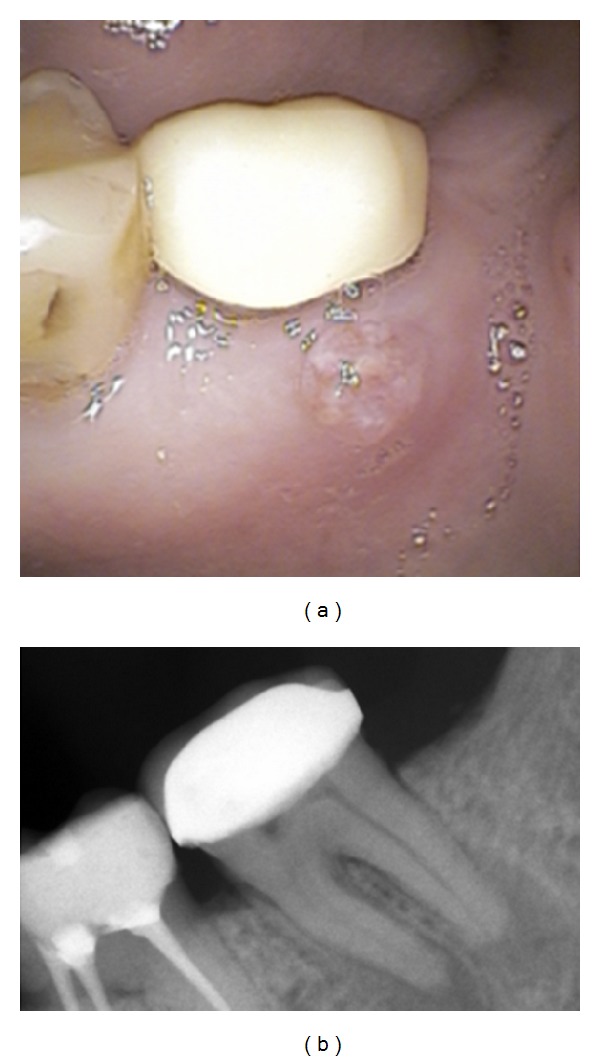
(a) Clinical image. Chronic suppurated apical periodontitis of tooth 37. Notice the fistula in the buccal area of this molar. (b) Radiological image. Notice the periapical radiolucent lesion in tooth 37.

**Figure 2:**
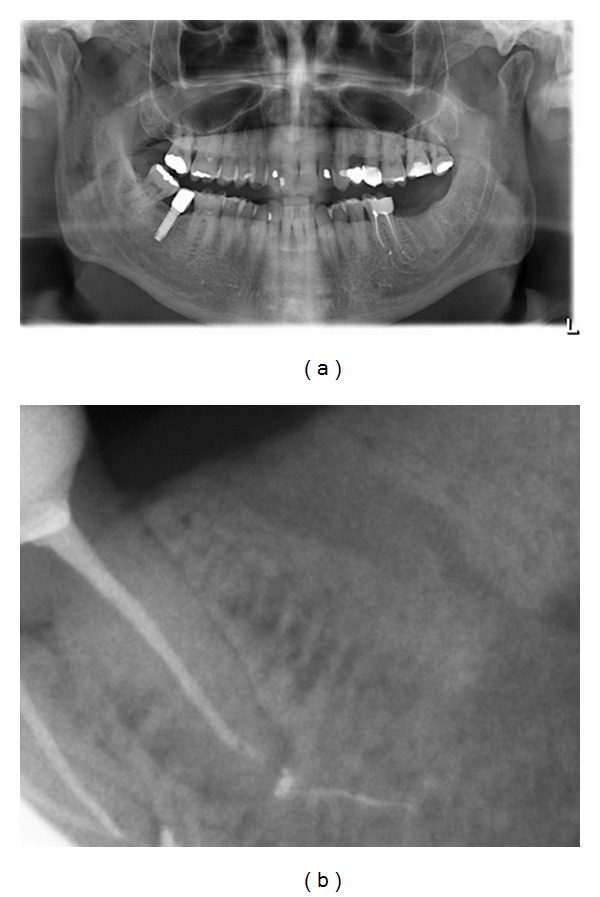
(a) Panoramic radiograph. Notice the correct healing of the socket after 6 months of the molar 37 extraction. (b) Periapical radiograph. Observe the satisfactory bone density in the socket after 6 months of extraction.

A 64-year-old Caucasian male was attended at Clinicians Associates in Terrassa (Barcelona); the chief complaint was a fistula on buccal area of tooth 37. At that time the patient was diagnosed with multiple myeloma stage III asymptomatic and only 2 doses of zoledronic acid (4 mg), once per month, have been administrated via IV. After a thorough clinicoradiological examination, a chronic suppurated apical periodontitis of tooth 37 was confirmed (Figures 1(a) and 1(b)). Considering that the analytical parameters were rather good and in order to avoid any active infectious lesion before proceeding to a bone marrow autotransplantation the extraction of this second molar was indicated. A couple of days before performing the conventional tooth extraction, antibiotic prophylaxis with Amoxicillin 500 mg/8 h and Metronidazole 500 mg/8 h was prescribed for 7 more days. The healing process had a complete satisfactory evolution and one week later sutures were removed. Two months later, bisphosphonate treatment was reintroduced with calcium supplementation and six months after the tooth extraction the clinicoradiological control of this localization was normal (Figure 2).

**Figure 3:**
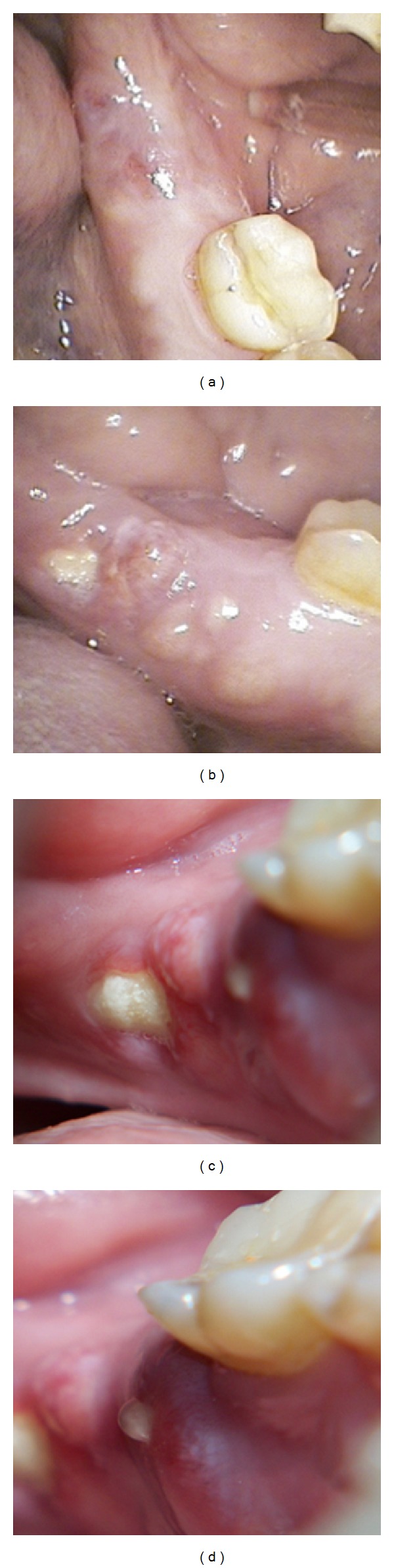
Clinical image. Progression of the lesion. (a) Notice the incipient mucositis and light tumefaction on the affected area. (b) Six weeks later, notice the bone exposure and how the lesion is expanding in mesial direction. ((c) and (d)) Eight weeks later, notice the increase of bone exposure and a characteristic sinus tract with its active exudation.

**Figure 4:**
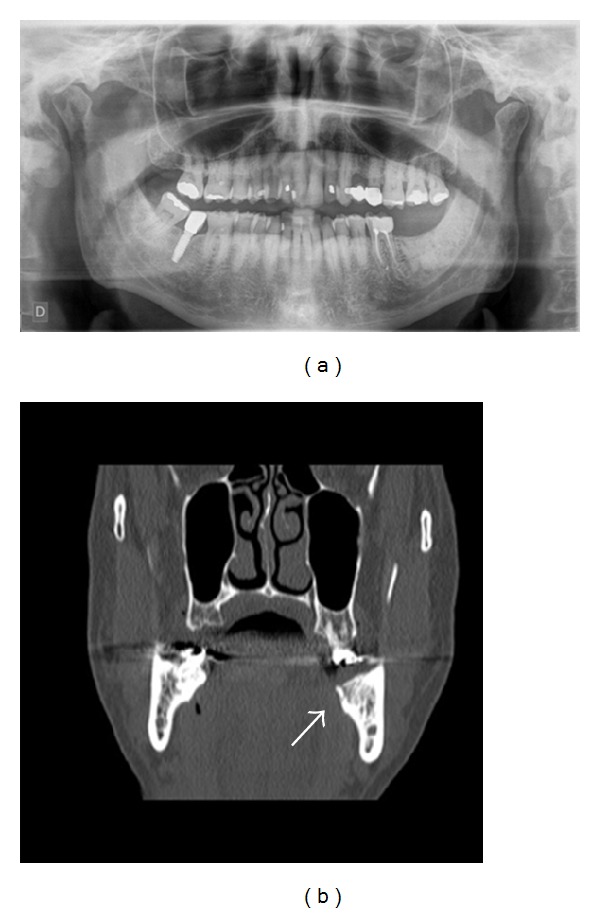
(a) Panoramic radiograph. Notice in the affected area a high bone density image. (b) CT image. Notice a lingual thin fissure line in the affected area.

**Figure 5:**
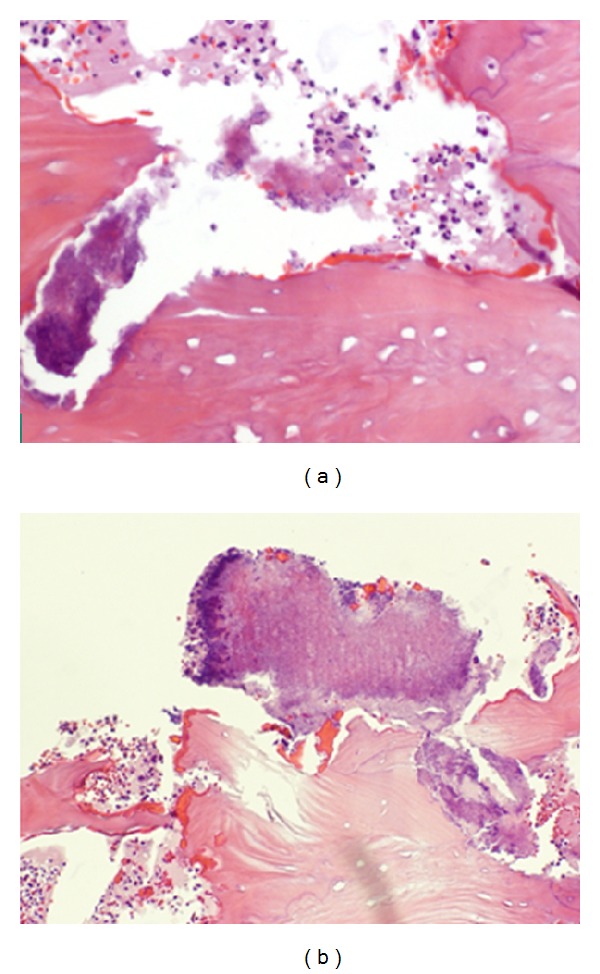
Microscopic appearance. (a) Necrotic bone fragment with acute inflammatory reaction with polymorphonuclear. H&E. Original magnification 20x. (b) Notice also the large bacterial aggregate consistent with* Actinomyces*. H&E. Original magnification 20x.

One and a half year after that tooth extraction the patient came for a routine control visit and he complained of slight pain in the left posterior alveolar ridge, in the lingual area. The patient mentioned that the discomfort started after a traumatic injury during mastication. Although radiologically there was not any relevant findings, clinically only one incipient point of inflamed mucosa was observed, which seemed like a simple foreign body reaction. Chlorhexidine topical gel was indicated and this lesion was monitored every two or three weeks also by the oral and maxillofacial surgeon in the Mútua Terrassa Hospital. The lesion size increased and an osteonecrosis of the jaw was the presumptive diagnosis. According to the clinical features and the presence of a sinus tract (Figures 3(a)–3(d)), antibiotic treatment was initiated, Amoxicillin 500 mg/8 h. In that moment the patient had completed his proposed bisphosphonate therapy after receiving the 6th dose of zoledronic acid (4 mg) via IV. A new panoramic radiography and a mandible computerized tomography (CT) were performed. The radiological report was not conclusive for osteonecrosis images, and only a lingual thin fissure line was observed in the affected area among a high bone density image (Figure 4). However, after 2-month follow-up the biopsy was indicated and its histopathological analysis confirmed the final diagnosis: osteonecrosis with* Actinomyces* infection (Figures 5(a) and 5(b)).

Despite a transitory clinical improvement of the lesion, its size was growing. The bone exposed area was greater concurring with the systemic corticosteroids administration in order to treat an acute exacerbation of the multiple myeloma. In a few days the patient general condition became worse over time and his toxic syndrome evolution was aggravated. About three weeks after the jaw lesion biopsy the patient was admitted to the hospital emergencies with severe hemogram alterations (leukocytes 1.66 × 10^9^/L, erythrocytes 3.1 × 10^12^/L, haemoglobin 9 gr/dL, and platelets 54 × 10^9^/L). Finally, a pneumonia due to* Pseudomonas aeruginosa* as an eventual complication of his pancytopenia and a massive haemoptysis episode precipitated his death.

## 3. Discussion

Usually the ONJ is a well-established bone disorder in patients treated with bisphosphonates that develops exposed necrotic bone in the oral cavity [14–16]. First report of a series of ONJ cases associated with bisphosphonates was published by Marx in 2003 [14].

There are two major theories regarding the pathophysiology of bisphosphonate-related ONJ. The osteoclast-based, “inside-out” theory, in which the inhibition of osteoclastic activity and marked suppression of bone turnover, together with spread of physiologic microdamage and possibly local infection, leads to bone death within the jaw, with subsequent exposure. The “outside-in” theory suggesting a break in the oral mucosa leads to ingress of bacteria and local infection which, coupled with poor bone remodelling, conduce to bone death. Bisphosphonate-related ONJ may result from combination of these two mechanisms and hypovascularity also plays an important role [17, 18]. In our case the initial minor traumatic injury might be the starting point of an “outside-in” process.

The risk of developing bisphosphonate-related osteonecrosis of the jaw associated with oral bisphosphonates, although exceedingly small, appears to increase when the duration of therapy exceeds 3 years. The time of exposure is a crucial factor for the development of ONJ [19]. It has been suggested that development of bisphosphonate-related ONJ requires a long period of exposure [16]. Bamias et al. observed that no patient who received fewer than 13 intravenous treatments with bisphosphonates developed osteonecrosis [20]. The authors observed that patients who developed ONJ received a median number of 35 infusions and the cumulative hazard increased above 1% after 12 months of treatment with bisphosphonates up to 11% at 4 years [20]. Similar observations have been reported by Durie et al. [21, 22]. In our reported clinical case, it is important to notice that when the tooth extraction was performed, only 2 doses of bisphosphonates had been administrated so it is rather unlikely a primary cause-effect with this drug. In addition the alveolar socket after extraction healed successfully. Although 18 months later a new process started by a minor traumatic injury to this area, when the patient had just received 6 infusions of bisphosphonates, the majority of bisphosphonates-related ONJ cases described in the literature highlight that the lesion is due to an unsatisfactory alveolar socket healing process after tooth extraction.

The clinical diagnosis of ONJ is usually made on the basis of visual inspection and/or radiographic examination. Panoramic and tomographic imaging may be performed to rule out other entities, like cysts, impacted teeth, or metastatic disease. The radiographic signs suggestive of ONJ involve osteolysis consistent with bone loss, providing a radiographic appearance similar to that observed in bone metastasis. Initially, minimal detectable radiographic changes are observed [1]. Contrarily, in our case report a high bone density was observed in the extraction socket. Such radiological feature could allow us to consider different but concomitant aetiological factors in this ONJ process.

ONJ may remain asymptomatic for many weeks or months and is usually identified by its unique clinical presentation of exposed bone in the oral cavity. Signs and symptoms of ONJ include localized discomfort, pain, soft-tissue swelling and inflammation, loosening of previously stable teeth, drainage, and exposed bone. These symptoms most commonly occur at the site of previous tooth extraction or other dental surgical interventions but may occur spontaneously. Other atypical complaints are the feeling of “heavy jaw,” “numbness,” and dysesthesia [1, 23, 24].

MM is characterized by osteolytic bone disease. The patients affected by this incurable plasma-cell malignancy present some additional risk factors, including the underlying malignancy, chemotherapy, corticosteroids, and systemic or regional infection [7]. Pancytopenia also increases the risk for infection and development of osteomyelitis. Vascular insufficiency due to thrombosis has been associated mechanistically with the development of the ONJ [25]. This event is a consequence of diminished arterial flow, increased intraosseous venous pressure, and osseous hypoxia [26]. Coagulopathies, typically manifested as thrombophilia or hypofibrinolysis, have been implicated as contributory factors [27] as has impaired blood flow secondary to edema or osteomyelitis [28–30]. So in our case the concomitant MM might have played an important role in the evolution of this ONJ. On the other hand, the massive hemoptysis episode before his demise would prove his severe coagulopathy.

Staging system of MM quantifies the extent of the disease in the body. The most commonly used system since 1975 has been the Durie-Salmon (DS) staging system [31–34]. In 2005 a new international method was utilized for myeloma prognosis based on the clinical and laboratory data. The International Staging System (ISS) utilizes a combination of serum *β*_2_ microglobulin and serum albumin to provide a simple, powerful, and reproducible three-stage classification (Table 1).

According to Ruggiero et al., tissue biopsy should be performed if metastatic disease is suspected. It has suggested a microbial culture (aerobic and anaerobic), to identify pathogens with the potential to cause secondary infections. It is important to note that* Actinomyces* organisms are often present [1].

Bisphosphonate-related ONJ was originally believed to be a direct, noninfectious complication of bisphosphonate therapy. However, recent histological and microbiological data strongly support that* Actinomyces* play a critical role in the pathogenesis of this disorder [35–40].* Actinomyces *spp. do not cause disease as long as they stay on the surface of the mucosa. However, if the integrity of the mucosal barrier is endangered and the bacteria gain access to the oral tissues or jawbones, they may initiate a prolonged chronic inflammatory process, creating a tumor-like mass, tissue destruction, osteolysis, and multiple sinus tracts [41–43]. During tissue invasion,* Actinomyces* form clumps called sulphur granules [44]. These* Actinomyces* bacterial aggregates were also identified in the microscopic analysis of our patient's biopsy (Figure 5).

The bisphosphonates were hypothesized to impede the repair process, resulting in avascular osteonecrosis [45–47]. However, other observations put this hypothesis into question and suggested that bisphosphonate use facilitates actinomycotic infection, resulting in osteonecrosis. Osteonecrosis solely due to bisphosphonates has not been described in animals or humans with normal bone [44] and the concomitant occurrence of* Actinomyces* infection with bisphosphonate-related ONJ has been seen in an increasing number of cases [35–40].

Management of ONJ has centred on efforts to eliminate or reduce severity of symptoms, to slow or prevent the progression of disease, and to eradicate diseased bone. Specific management regimens have included chlorhexidine rinses, antibiotic therapy, nonsurgical sequestrectomy (simple removal of mobile bone fragments), and surgical debridement and/or resection of necrotic bone [8].

American Academy of Oral and Maxillofacial Surgeons (AAOMS) adopted stage system guidelines designed to minimize symptoms and/or achieve resolution of lesions (Table 2). Regardless of stage, chlorhexidine rinses were prescribed for the majority of patients and mobile fragments of bone were managed with nonsurgical sequestrectomy, typically without the need for local anaesthesia. Asymptomatic patients were typically managed with observation generally including chlorhexidine. Symptomatic patients were generally treated with antibiotics for management of pain or the presence of sinus tracts until these resolved. Surgical debridement, resection, and/or reconstruction were reserved for severe cases and/or disease refractory at previous measures. Some authors recommend staging the patients at the initial visit and at each subsequent visit, in order to evaluate the course and management outcomes [8].

Van den Wyngaert et al. observed that a combination of antimicrobial rinses, antibiotic therapy, nonsurgical sequestrectomy, and local debridement is an appropriate and effective approach for management of ONJ [48].

The goal of the intermittent or continuous antibiotic therapy is to prevent secondary soft-tissue infection, pain, and osteomyelitis. Microbial cultures should be collected and analyzed to determine the appropriate antimicrobial intervention. In some refractory cases, patients might require combination antibiotic therapy, long-term antibiotic maintenance, or course of IV antibiotic therapy [8]. Several antimicrobial pharmacologic therapies have been recommended (Table 3).

## 4. Conclusion

This clinical case shows a singular presentation of an ONJ in a patient with multiple myeloma-bone disease and other concomitant factors. However, the traumatic mechanism “outside-in” was the most probable origin of the lesion, as the tooth extraction was performed 18 months ago with a satisfactory healing. Moreover, the few doses of bisphosphonates administrated might also support the infectious aetiology, caused by* Actinomyces*, as a starting point.

## Figures and Tables

**Table 1:** International Staging System (ISS) for multiple myeloma.

Stage	Criteria
I	Serum *β*_2_ microglobulin < 3.5 mg/L and serum albumin ≥ 3.5 g/dL

II	Serum *β*_2_ microglobulin < 3.5 mg/L but serum albumin < 3.5 g/dL or serum *β*_2_ microglobulin 3.5 to < 5.5 mg/L irrespective of the serum albumin level

III	Serum *β*_2_ microglobulin ≥ 5.5 mg/L

**Table 2:** ONM staging and treatment strategies—American Association of Oral and Maxillofacial Surgeons 2009.

ONJ stage	Description	Treatment strategies
At risk category	No apparent necrotic bone in patients who have been treated with either oral or IV bisphosphonates	No treatment indicated Patient education

Stage 0	No clinical evidence of necrotic bone, but nonspecific clinical findings and symptoms	Systemic management, including use of pain medication and antibiotics

Stage 1	Exposed and necrotic bone in asymptomatic patients without evidence of infection	Antibacterial mouth rinse Clinical follow-up on quarterly basis Patient education and review of indications for continued bisphosphonate therapy

Stage 2	Exposed and necrotic bone associated with infection as evidenced by pain and erythema in region of exposed bone with or without purulent drainage	Symptomatic treatment with oral antibiotics Oral antibacterial mouth rinse Pain control Superficial debridement to relieve soft tissue irritation

Stage 3	Exposed and necrotic bone in patients with pain, infection, and one or more of the following: A—exposed and necrotic bone extending beyond the region of alveolar bone, (i.e., inferior border and ramus in the mandible or maxillary sinus and zygoma in the maxilla) resulting in pathologic fracture, B—extraoral fistula and oral antral/oral nasal communication, and C—osteolysis extending to the inferior border of the mandible or the sinus floor	Antibacterial mouth rinse Antibiotic therapy and pain control Surgical debridement/resection for longer term palliation of infection and pain.

**Table 3:** Anti-infective pharmacologic treatments∗.

Treatment	Dose and schedule
Antibacterials	
Penicillin VK	500 mg every 6 to 8 hours for 7 to 10 days and then every 12 hours for maintenance
Amoxicillin	500 mg every 8 hours for 7 to 10 days and then every 12 hours for maintenance
Patients with penicillin allergy	
Clindamycin	150 to 300 mg every 6 hours
Vibramycin	100 mg every 24 hours
Erythromycin ethylsuccinate	400 mg every 8 hours
Azithromycin	500 mg PO × 1 on day 1; 250 mg oral every 6 hours on days 2 to 5
Antifungals^†^ (when required)	
Nystatin oral suspension	5 to 15 mL every 6 hours or 100.000 IU/mL
Clotrimazole	10 mg every 8 hours and every 5 hours on days 7 to 10
Fluconazole	200 mg initially and then 100 mg every 24 hours
Antivirals^‡^	
Acyclovir	400 mg every 12 hours
Valacyclovir hydrochloride	500 mg to 2 g every 12 hours

^†^Other potential systemic antifungals include itraconazole or ketoconazole.

^‡^Role of antivirals in the treatment of osteonecrosis of the jaw has not yet been established.

∗Novartis (Basel, Switzerland), data on file.
